# Examination of a pre-exercise, high energy supplement on exercise performance

**DOI:** 10.1186/1550-2783-6-2

**Published:** 2009-01-06

**Authors:** Jay R Hoffman, Jie Kang, Nicholas A Ratamess, Mattan W Hoffman, Christopher P Tranchina, Avery D Faigenbaum

**Affiliations:** 1 Department of Health and Exercise Science, The College of New Jersey, PO Box 7718, Ewing, New Jersey 08628, USA

## Abstract

**Background:**

The purpose of this study was to examine the effect of a pre-exercise high energy drink on reaction time and anaerobic power in competitive strength/power athletes. In addition, the effect of the pre-exercise drink on subjective feelings of energy, fatigue, alertness and focus was also explored.

**Methods:**

Twelve male strength/power athletes (21.1 ± 1.3 y; 179.8 ± 7.1 cm; 88.6 ± 12.1 kg; 17.6 ± 3.3% body fat) underwent two testing sessions administered in a randomized and double-blind fashion. During each session, subjects reported to the Human Performance Laboratory and were provided with either 120 ml of a high energy drink (SUP), commercially marketed as Redline Extreme^® ^or 120 ml of a placebo (PL) that was similar in taste and appearance but contained no active ingredients. Following consumption of the supplement or placebo subjects rested quietly for 10-minutes prior to completing a survey and commencing exercise. The survey consisted of 4 questions asking each subject to describe their feelings of energy, fatigue, alertness and focus for that moment. Following the completion of the questionnaire subjects performed a 2-minute quickness and reaction test on the Makoto testing device (Makoto USA, Centennial CO) and a 20-second Wingate Anaerobic Power test. Following a 10-minute rest subjects repeated the testing sequence and after a similar rest period a third and final testing sequence was performed. The Makoto testing device consisted of subjects reacting to both a visual and auditory stimulus and striking one out of 30 potential targets on three towers.

**Results:**

Significant difference in reaction performance was seen between SUP and PL in both average number of targets struck (55.8 ± 7.4 versus 51.9 ± 7.4, respectively) and percent of targets struck (71.9 ± 10.5% versus 66.8 ± 10.9%, respectively). No significant differences between trials were seen in any anaerobic power measure. Subjective feelings of energy (3.5 ± 0.5 versus 3.1 ± 0.5) and focus (3.8 ± 0.5 versus 3.3 ± 0.7) were significantly higher during SUP compared to PL, respectively. In addition, a trend towards an increase in average alertness (p = 0.06) was seen in SUP compared to P.

**Conclusion:**

Results indicate a significant increase in reaction performance, with no effect on anaerobic power performance. In addition, ingestion of this supplement significantly improves subjective feelings of focus and energy in male strength/power athletes.

## Background

High energy drinks and capsules have recently been shown to be the most popular supplement besides multivitamins in the American adolescent and young adult population [1,2]. More than 30% of all American male and female adolescents are reported to use these supplements on a regular basis. The primary reason for use of these supplements is thought to be related to their desire to reduce or control body fat [1-4]. However, many athletes use these high energy supplements for its potential ergogenic effect. They believe that using high energy supplements prior to performance will result in greater focus, reaction time and power. Unfortunately, most information available is based upon empirical evidence.

Several papers have been published showing that a pre-exercise, high energy supplement can delay fatigue and/or improve the quality of a resistance training workout [5-7]. A recent study showed that a pre-exercise high energy supplement consumed 10 minutes before resistance exercise can enhance acute exercise performance by increasing the number of repetitions performed and the total volume of exercise [7]. The enhanced exercise performance resulted in a significantly greater increase in both growth hormone and insulin concentrations, indicating an augmented anabolic hormone response to this pre-exercise supplement. Although the ergogenic benefits associated with high energy supplements have been demonstrated, the ability to improve subjective feelings of focus, awareness or improve reaction time is not clear. Anecdotal reports suggest that many athletes use high energy supplements prior to an athletic contest to enhance these specific components. However, studies examining the ability of these pre-exercise energy supplements to improve reaction time and performance are scarce.

Many pre-exercise high energy supplements consist of multiple ingredients that are proposed to either increase metabolic rate, enhance exercise performance or both. One such supplement is known as Redline Extreme™. It consists of various herbal and amino acid ingredients which include evodiamine, vinpocetine, yohimbine, hordenine, salbutiamine, beta-alanine, tyrosine, and tyramine. These herbs and amino acids are suggested to work synergistically to enhance exercise performance. Thus, it is the purpose of this study to examine the effect of a popular, over-the-counter high energy supplement on physical performance and subjective feelings of energy, focus, awareness and fatigue in strength/power athletes.

## Methods

### Subjects

Twelve male strength/power athletes (mean ± SD; 21.1 ± 1.3 y; 179.8 ± 7.1 cm; 88.6 ± 12.1 kg; 17.6 ± 3.3% body fat) volunteered for this study. Following an explanation of all procedures, risks and benefits each subject gave his written informed consent to participate in this study. The Institutional Review Board of The College of New Jersey approved the research protocol. Subjects with any known metabolic or cardiovascular disease, or psychiatric disorder were excluded. Subjects were also required to have been free of any nutritional supplements or ergogenic aids for the 6 weeks preceding the study, and were asked to refrain from taking any additional supplement during the duration of the study.

### Study design

The study followed a randomized double-blind, crossover design. Subjects reported to the Human Performance Laboratory on two separate days. Each testing session was separated by one week. Subjects were instructed to refrain from consuming any caffeine products on the day of each testing session and from performing any strenuous physical activity for the previous 12 hours. In addition, subjects were instructed not to eat or drink for 3 hours prior to each trial. Following a 10 min resting period subjects were randomly provided with either the supplement (SUP) or the placebo (PL). On the subject's second visit to the laboratory they were provided with the opposite treatment.

**Figure 1:**
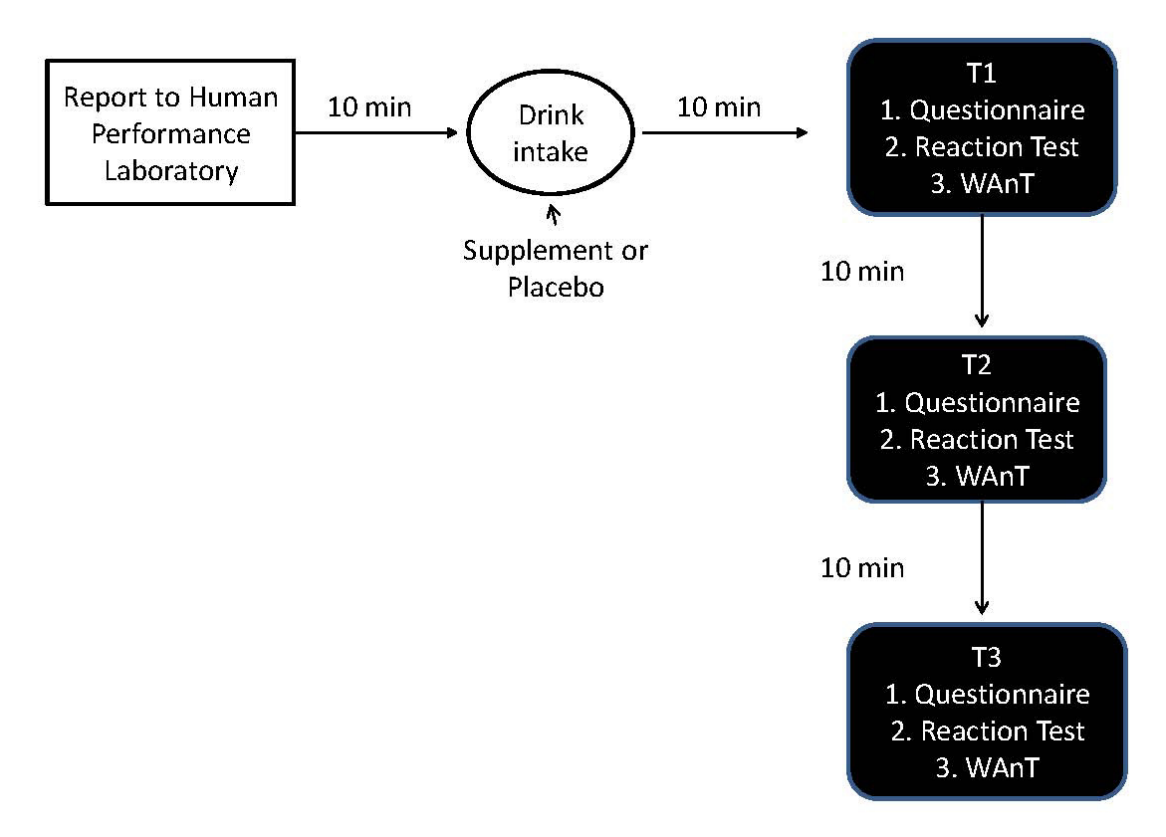
**Study Protocol**. WAnt = Wingate Anaerobic Power Test.

Following consumption of the supplement or placebo subjects, rested quietly for 10-minutes prior to completing a survey and commencing exercise (T1). The survey consisted of 4 questions asking each subject to describe their feelings of energy, fatigue, alertness and focus for that moment. Following the completion of the questionnaire subjects performed a 2-minute quickness and reaction test on the Makoto testing device (Makoto USA, Centennial CO) and a 20-second Wingate Anaerobic Power test. Following a 10-minute rest subjects repeated the testing sequence (T2) and after a similar rest period a third and final testing sequence was performed (T3). The study protocol is depicted in Figure 1.

### Reaction test

**Figure 2:**
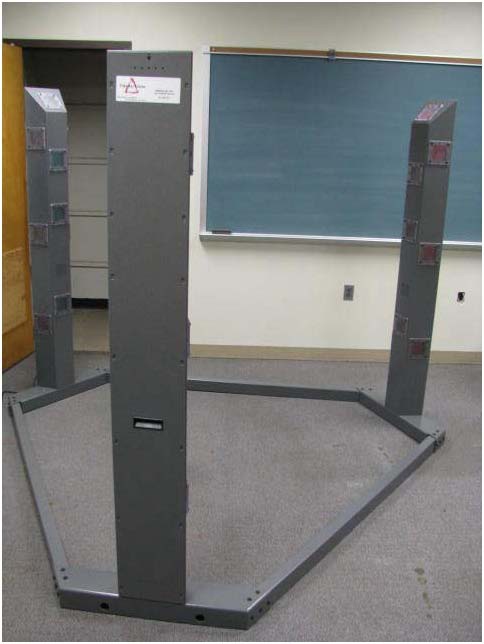
**Makoto Testing Device**.

The measure of reaction time was assessed using the Makoto testing device (Makoto USA, Centennial CO). The Makoto device is in the shape of a triangle that is eight feet from base to apex (see Figure 2). It consists of three steel towers that are six feet high. Each tower contains ten targets. For each test the subject stood in the middle of the triangle holding a padded staff with both hands and faced one of the towers with the other two in his peripheral vision. The reaction test began with a loud auditory stimulus. During the next two minutes subjects were required to react to both a visual (targets light up) and auditory (loud gong) stimulus. As the gong sounded and the light on the target lit up the subject was required to lunge and make contact with the target using the staff. Subjects had to make contact to the target prior to the light and sound stopping. If the subject made contact with the target within the required time it was registered as a 'hit'. Subjects were required to make as many contacts as possible within the 2-min period. A total of three trials were conducted (one trial during each 10 min period) and the average number of hits was determined and the average percentage of hits [(successful contacts/total number of possible stimuli)*100] was calculated.

The Makoto testing device has 12 levels of skill. All tests for this study were conducted at the highest level (level 12). All subjects completed familiarization sessions prior to entering the study. All familiarization sessions started at level 7. To advance to the next level subjects needed to be within 10% of their score for two consecutive trials (plateau effect). Advancements were made two levels at a time. For instance, subjects performed familiarization sessions at levels 7, 9 and 11. Subjects performed on average 9.5 ± 1.9 familiarization sessions.

### Anaerobic power measure

To quantify anaerobic power performance all subjects performed a modified Wingate anaerobic power test (Lode Excalibur, Groningen, The Netherlands). After a warm-up period of 5 min of pedaling at 60 rpm interspersed with an all-out sprint lasting 5 s, the subjects pedaled for 20 s at maximal speed against a constant force (1.2 Nm·kg^-1^). Peak power, mean power, time to peak power, total work and a fatigue index was determined. Peak power was defined as the highest mechanical power output elicited during the test. Mean power was defined as the average mechanical power during the 20 s test. The fatigue index was determined by dividing the highest power output by the lowest power output. A total of three 20-s Wingate tests (one Wingate test per 10-min period) were performed during each trial and measures were averaged over the three sprints.

### Questionnaires

Prior to each bout of performance measures subjects were asked to complete a questionnaire containing four questions using a 5-point rating scale. Subjects were asked to rate their energy level, fatigue level, feelings of alertness and feelings of focus for task using the following verbal anchors: 1 = very low; 2 = low; 3 = average; 4 = high; 5 = very high. The same researcher performed all test administrations and tests were conducted under controlled conditions (a quiet room). The average response of the three testing sessions was computed.

### Supplement

On each visit subjects consumed 120 ml of the ready to drink supplement or placebo. The supplement used is marketed as Redline Extreme^® ^(Vital Pharmaceuticals, Davie, FL) and contains caffeine anhydrous, beta-alanine, vitamin C, and the following herbal and botanical compounds; evodiamine, N-acetyl-L-tyrosine, hordenine, 5-hydroxytryptophan, potassium citrate, N-methyl tyramine, sulbutiamine, vinpocetine, yohimbine HCL, and St. John's wort extract. The placebo was similar in appearance and taste to Redline Extreme^®^, but contained only an inert substance.

### Statistical analyses

Statistical analysis of the data was accomplished using a repeated measures analysis of variance. In the event of a significant F-ratio, LSD post-hoc tests were used for pairwise comparisons. Comparisons of the average performance measures for the three testing periods were analyzed using paired student's T-tests. A criterion alpha level of p ≤ 0.05 was used to determine statistical significance. All data are reported as mean ± SD.

## Results

The responses to the questionnaire can be seen in Table 1. The average energy level during the three testing periods was significantly higher for SUP than PL. In addition, focus for task was significantly greater at T3 for SUP than PL, and the average focus for task for all three testing periods combined was significantly higher for SUP than PL. Average feelings of alertness tended to be higher (p < 0.06) for SUP than PL. No significant differences in perceived levels of fatigue were seen between the groups.

**Table 1:** Response to performance questionnaire

**Question**	**Group**	**T1**	**T2**	**T3**	**AVG**
My energy level is:	Sup	3.7 ± 0.7	3.5 ± 0.7	3.3 ± 0.6	3.5 ± 0.5 *
	
	PL	3.2 ± 0.6	3.2 ± 0.6	2.8 ± 0.9	3.1 ± 0.5

My fatigue level is:	Sup	2.3 ± 0.9	2.8 ± 0.8	3.1 ± 0.7	2.7 ± 0.6
	
	PL	2.4 ± 0.7	3.1 ± 0.5	3.3 ± 0.9	2.9 ± 0.5

My feeling of alertness is:	Sup	3.7 ± 0.7	3.6 ± 0.5	3.6 ± 0.7	3.6 ± 0.4
	
	PL	3.3 ± 0.7	3.4 ± 0.7	3.1 ± 1.0	3.3 ± 0.7

My feeling of focus for task is:	Sup	3.8 ± 0.7	3.8 ± 0.6	3.7 ± 0.7 *	3.8 ± 0.5 *
	
	PL	3.3 ± 0.7	3.5 ± 0.7	3.0 ± 1.0	3.3 ± 0.7

SUP = Supplement; PL = Placebo; * = significant difference between SUP and PL. All data are reported as Mean ± SD.

**Figure 3:**
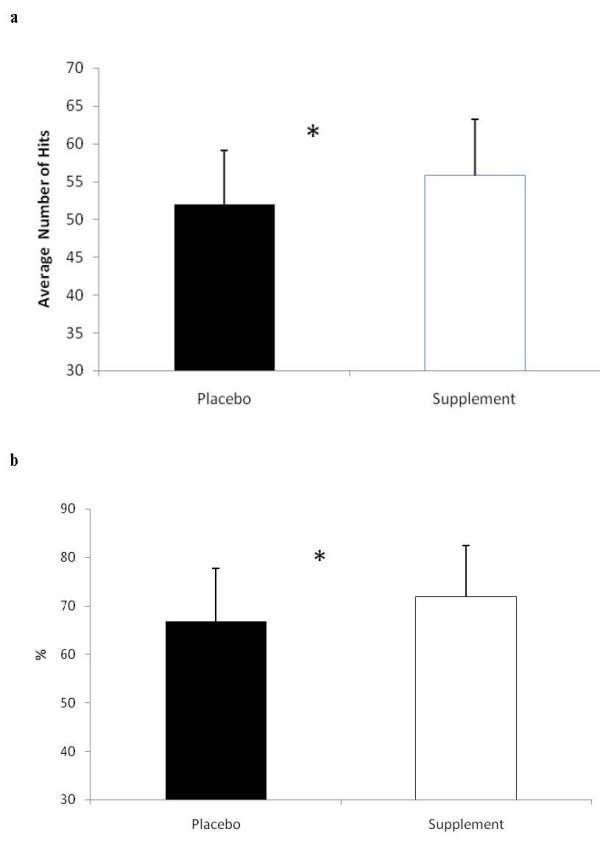
**a: Reaction time: Average number of hits**. * = Significant difference (p < 0.05) between the supplement and placebo. **b**: Reaction time: Average percentage of successful hits from total possible targets. * = Significant difference (p < 0.05) between the supplement and placebo. Data are reported mean ± SD.

The anaerobic power measures are depicted in Table 2. No between group differences in any performance measure were seen at any testing period and no differences in the average performance measure were seen between SUP and PL. Significant differences in reaction time were seen between the groups. The average number of successful hits to target was significantly higher for SUP than PL (see Figure 3a), and the average percentage of successful hits on target was also significantly greater for SUP than PL (see Figure 3b)

**Table 2:** Anaerobic power measures

**Variable**	**Group**	T1	T2	T3	AVG
Mean Power (W)	Sup	665 ± 19	675 ± 27	686 ± 35	675 ± 23
	
	PL	671 ± 32	684 ± 36	684 ± 43	680 ± 33

Mean Power (W/kg)	Sup	7.7 ± 1.1	7.8 ± 1.2	7.9 ± 1.2	7.8 ± 1.2
	
	PL	7.7 ± 1.1	7.9 ± 1.2	7.8 ± 1.1	7.8 ± 1.1

Peak Power (W)	Sup	1099 ± 107	1097 ± 107	1098 ± 113	1098 ± 101
	
	PL	1094 ± 76	1085 ± 111	1075 ± 120	1084 ± 95

Peak Power (W/kg)	Sup	12.4 ± 1.4	12.0 ± 1.2	12.1 ± 1.7	12.2 ± 1.3
	
	PL	12.5 ± 1.4	12.3 ± 1.2	12.2 ± 1.5	12.3 ± 1.3

Time to Peak Power (Sec)	Sup	4.0 ± 0.1	4.0 ± 0.1	4.3 ± 1.0	4.1 ± 0.3
	
	PL	4.1 ± 0.4	4.2 ± 0.6	4.2 ± 0.4	4.2 ± 0.3

Rate of Fatigue (W/sec)	Sup	31.1 ± 5.5	30.5 ± 7.4	29.5 ± 8.4	30.4 ± 6.4
	
	PL	31.0 ± 4.9	30.9 ± 5.8	31.2 ± 6.1	31.1 ± 5.0

Total Work (J)	Sup	13300 ± 401	13500 ± 546	13713 ± 694	13515 ± 468
	
	PL	13432 ± 599	13678 ± 719	13683 ± 861	13598 ± 651

SUP = Supplement; PL = Placebo; All data are reported as Mean ± SD.

## Discussion

The results of this study indicate that a pre-exercise energy drink containing anhydrous caffeine, beta-alanine, vitamin C, evodiamine, N-acetyl-L-tyrosine, hordenine, 5-hydroxytryptophan, potassium citrate, N-methyl tyramine, sulbutiamine, vinpocetine, yohimbine HCL, and St. John's wort extract can significantly improve reaction time and enhance self-perceived feelings of energy and focus. In addition, a trend towards improved alertness in subjects using this supplement versus placebo was also seen. Supplement ingestion did not have any ergogenic benefit for anaerobic power performance.

Caffeine is a mild central nervous system stimulant, whose effects are similar to those associated with amphetamines, only much weaker [8]. It has been used as an ergogenic aid for many years, but consistent benefits have only been seen during exhaustive endurance exercise in which time to exhaustion is often improved [5,9-11]. This is thought to be related to caffeine's ability to enhance reliance on fat oxidation preserving muscle glycogen content [12]. Although caffeine has been suggested to augment strength and power performance by enhancing excitation – contraction coupling during neuromuscular transmission through mobilizing intracellular calcium ions from the sarcoplasmic reticulum [13] and/or by enhancing the kinetics of glycolytic regulatory enzymes such as phosphorylase [12], evidence demonstrating its ergogenic benefit during anaerobic performance is limited.

To maximize the effectiveness of caffeine, supplements often contain several ingredients that attempt to exacerbate its stimulatory potential. The combination of ephedra and caffeine had been shown to be an effective ergogenic aid [14], however, the multitude of adverse events associated with ephedra led to this supplement being removed from the sport supplement market [15,16]. As a result, other ingredients that stimulate β-adrenergic receptors albeit with a lower risk for adverse events have been combined with caffeine with the desire to enhance athletic performance either by improving metabolic or muscle contraction efficiency, or perhaps by enhancing subjective feelings of energy, focus or awareness. The results of this study indicate that the combination of ingredients comprising Redline Extreme™ were effective in providing a greater stimulatory response as reflected by higher self-perceived levels of focus, energy and awareness and an enhanced reaction to visual and audio stimuli. The combination of these stimulatory ingredients though was unable to augment anaerobic power performance.

The combination of yohimbine, evodiamine, hordenine, tyramine, tyrosine and caffeine appear to be the primary ingredients providing the stimulatory effect from Redline Extreme^®^. Yohimbine is a selective α-adrenoceptor antagonist that is also reported to be effective in enhancing lipid metabolism [17,18]. Evodiamine is a major alkaloid from evodia fruits that has been reported to stimulate vanilloid receptor activities comparable to capsaicin (compound found in hot peppers) [19]. Research on evodiamine is limited, but it has been shown to increase core body temperature [20]. Hordenine is also an alkaloid and is found in grains, sprouting barley and certain grasses, as well as in small quantities in citrus aurantium [21]. Citrus aurantium is a mild stimulant that is often used in nutritional supplements to suppress appetite and enhance metabolic rate [22]. Tyramine is a monoamine compound that is derived from the amino acid tyrosine. It is an indirect sympathomimetic, meaning that it does not directly activate adrenergic receptors, but acts as a substrate for adrenergic uptake systems and monoamine oxidase prolonging the actions of adrenergic transmitters [23]. Tyrosine is a precursor for the synthesis of dopamine and norepinephrine [24]. Its role is to enhance neurotransmitter synthesis that has important β-adrenergic stimulatory effect. Although the combination of these compounds in this supplement appears to be effective in producing the stimulatory effect as demonstrated presently, the proprietary nature (e.g., precise concentration of each ingredient is not released) of this supplement limits discussion on the possible extent of contribution from each ingredient.

Sulbutiamine is a centrally acting cholinergic agent that has been shown to be effective in treating fatigue or central weakness in clinical populations [25,26]. Its efficacy in young, athletic populations is not known, and this appears to be the first study to examine its efficacy for enhancing energy in this subject population. Vinpocetine is a derivative of vinacamine; a purified extract of *Vinca Minor L *(Periwinkle plant). It has previously been used as a cerebral vasodilator for enhancing mental alertness and memory [27]. It is likely that the combination of these ingredients contributed to the enhanced energy and focus experienced by the subjects in this study.

The role that the additional ingredients in the supplement (e.g beta-alanine, 5-hydroxytryptophan and St Johns wort extract) may have played is not clear. Beta-alanine is a non-proteogenic amino acid that can enhance the buffering capacity of muscle by increasing muscle carnosine concentrations [28]. Its role as a high energy supplement though is questionable, considering that it has no known acute effect on metabolic rate or stimulation of adrenergic receptors [15]. The addition of 5-hydroxytryptophan and St John's wort extract as ingredients may be related to their potential for mood enhancement. 5-hydroxytryptophan is thought to enhance mood by stimulating dopamine release [29] and enhancing serotonin production [30], while St John's wort extract appears to act by reducing β-adrenergic receptor binding [31]. Although mood was not measured in this study, it is possible that these ingredients may have influenced the stimulatory effect of this supplement and contributed to the enhanced feelings of focus, energy and awareness that subsequently enhanced reaction time.

In conclusion, results of this study indicate that the supplement Redline Extreme^® ^can significantly improve subjective feelings of focus and energy leading to a significant increase in reaction time to both visual and auditory stimuli in strength/power athletes. However, acute ingestion of this supplement had no effect on anaerobic power performance.

## Competing interests

Vital Pharmaceuticals. (Davie, FL) provided funding for this project. All researchers involved collected, analyzed, and interpreted the results from this study and have no financial interests concerning the outcome of this investigation. Publication of these findings should not be viewed as endorsement by the investigators, The College of New Jersey or the editorial board of the Journal of International Society of Sports Nutrition.

## Authors' contributions

JRH was the primary investigator, obtained grant funds for project, designed study, supervised all study recruitment, data/specimen analysis, statistical analysis and manuscript preparation. JK, NAR, and ADF were co-authors, oversaw all aspects of study including recruitment, data/specimen analysis, and manuscript preparation. MWH, and CPT were co-authors, assisting with data collection and data analysis.
